# Rediscovery and redescription of the endangered *Hypostomus subcarinatus* Castelnau, 1855 (Siluriformes: Loricariidae) from the Rio São Francisco basin in Brazil

**DOI:** 10.1371/journal.pone.0207328

**Published:** 2019-03-12

**Authors:** Cláudio Henrique Zawadzki, Iago de Souza Penido, José Carlos de Oliveira, Tiago Casarim Pessali

**Affiliations:** 1 Universidade Estadual de Maringá. Departamento de Biologia. Núcleo de Pesquisas em Limnologia, Ictiologia e Aquicultura (Nupélia), Maringá, Paraná, Brazil; 2 Universidade Estadual de Maringá. Programa de Pós-Graduação em Biologia Comparada, Maringá, Paraná, Brazil; 3 Universidade Federal de Juiz de Fora. Instituto de Ciências Biológicas. Departamento de Zoologia, Rua José Lourenço Kelmer, Juiz de Fora, Minas Gerais, Brazil; 4 Museu de Ciências Naturais da PUC Minas, Belo Horizonte, Minas Gerais, Brazil; Pontificia Universidade Catolica do Rio Grande do Sul, BRAZIL

## Abstract

*Hypostomus subcarinatus* Castelnau, 1855 is rediscovered in the Lagoa da Pampulha, an urban lake in the Rio das Velhas basin (Rio São Francisco system) in the state of Minas Gerais, southeastern Brazil. Herein, *H*. *subcarinatus* is redescribed and diagnosed from its congeners based on characters such as blue-tan dorsal fin in live specimens, slender bicuspid teeth, dentaries angled more than 90 degrees, moderate keels along lateral series of plates, small roundish dark spots, one plate bordering supraoccipital, by having nuptial odontodes mainly on pectoral, dorsal and caudal-fin rays, and long anal-fin unbranched ray. The rediscovery of *H*. *subcarinatus* more than 160 years after its original description was an unexpected event, because the Lagoa da Pampulha is an artificial, silted and polluted urban lake. The lake is located in downtown Belo Horizonte, the third largest urban agglomeration in Brazil with a population exceeding 5.9 million inhabitants.

## Introduction

The loricariid *Hypostomus subcarinatus* was described by Castelnau [1] from a vague type locality stated as “des rivière de la province des Mines” [streams from the state of Minas Gerais]. Therefore, it was hypothetically associated with Eastern Brazilian coastal drainages and to the Rio São Francisco basin [2]. However, despite some ichthyological survey efforts in these systems [3,4], no scientific record of *H*. *subcarinatus* was undoubtedly stated for more than 160 years. This historical lack of records of *H*. *subcarinatus* lead to some hypotheses, a) an erroneous locality designation in the original description by Castelnau; b) species rarity or endemicity to specific locations; c) several ongoing populational extinction processes; or d) imprecise identifications.

In 2014 a fish environmental monitoring survey was conducted in the Lagoa da Pampulha, an artificial silted and polluted urban lake of the Rio São Francisco basin system and located in downtown Belo Horizonte, Minas Gerais State, southeastern Brazil. Unexpectedly, seven large specimens of the catfish *Hypostomus* were captured. Subsequent examination did not allow to recognize them as any of the commonly found species of *Hypostomus* of the Rio São Francisco basin. However, in comparison to *Hypostomus* original descriptions, as well as to type series of *Hypostomus* from worldwide scientific fish collections the specimens were recognized as Castelnau’s (1855) lost *Hypostomus subcarinatus*. In this work the species is redescribed and the importance of the conservation of urban water bodies is claimed.

## Material and methods

Fishes were collected under permits from the Instituto Chico Mendes de Conservação da Biodiversidade—ICMBio n. 9101-1/2017. Captured individuals were anaesthetized and sacrificed by immersion in eugenol (active ingredient: phenolic eugenol, 4-Allyl-2-methoxyphenol-C10H12O2, derived from stems, flowers and leaves of *Eugenia caryophyllata* and *Eugenia aromatica* trees) [5], fixed in 10% formalin solution and later preserved in 70% ethanol. These procedures are in accordance to the ‘Ethical Principles in Animal Research’ guidelines adopted by the National Council of Control of Animal Experimentation (CONCEA). Measurements and counts of bilaterally symmetrical features were taken from the left side of the body, whenever possible. Measurements were taken using a digital caliper to the nearest 0.1 mm. Methodology and terminology of measurements follows Boeseman [6], modified by Weber [7] and Zawadzki *et al*. [8]. Plate counts and bone nomenclature follow Schaefer [9], modified by Oyakawa *et al*. [10]. Standard length (SL) is expressed in millimeters and all other measurements are expressed as percent of standard length or head length (HL), unless otherwise noted. Institutional abbreviations of material deposited follow Fricke & Eschmeyer [11], except ICT-UFMG (Coleção Ictiológica do Centro de Coleções Taxonômicas do Instituto de Ciências Biológicas da Universidade Federal de Minas Gerais). The species conservation status was assessed through the criteria of the International Union for Conservation of Nature (IUCN standards and petitions subcommittees, 2017 [12]) guidelines.

## Results

### *Hypostomus subcarinatus* Castelnau, 1855

#### Diagnosis

*Hypostomus subcarinatus* is distinguished from all congeners by having a blue-tan dorsal fin in living specimens (vs. not having a blue-tan dorsal fin). Additionally, *H*. *subcarinatus* is diagnosed from the species of the *H*. *cochliodon* group by having slender viliform bicuspid teeth (vs. robust spoon-shaped teeth) and by having dentaries angled to each other more than 90 degrees (vs. dentaries angled from 80 to 90 degrees); from the remaining congeners except *H*. *affinis*, *H*. *ancistroides*, *H*. *argus*, *H*. *aspilogaster*, *H*. *borellii*, *H*. *boulengeri*, *H*. *carinatus*, *H*. *careopinnatus*, *H*. *commersoni*, *H*. *corantijni*, *H*. *crassicauda*, *H*. *delimai*, *H*. *dlouhyi*, *H*. *faveolus*, *H*. *formosae*, *H*. *gymnorhynchus*, *H*. *hemiurus*, *H*. *hoplonites*, *H*. *interruptus*, *H*. *micromaculatus*, *H*. *niceforoi*, *H*. *nigrolineatus*, *H*. *pantherinus*, *H*. *paucimaculatus*, *H*. *piratatu*, *H*. *plecostomus*, *H*. *punctatus*, *H*. *pusarum*, *H*. *rhantos*, *H*. *scabriceps*, *H*. *seminudus*, *H*. *tapijara*, *H*. *velhochico* and *H*. *watwata*, by having moderate keels along the five lateral series of plates (vs. lacking keels); from the species above by having a more slender and elongate body, what can be somewhat depicted by the anal-fin unbranched ray length longer than head depth (vs. anal-fin unbranched ray length shorter than head depth).

#### Description

**Fig 1:**
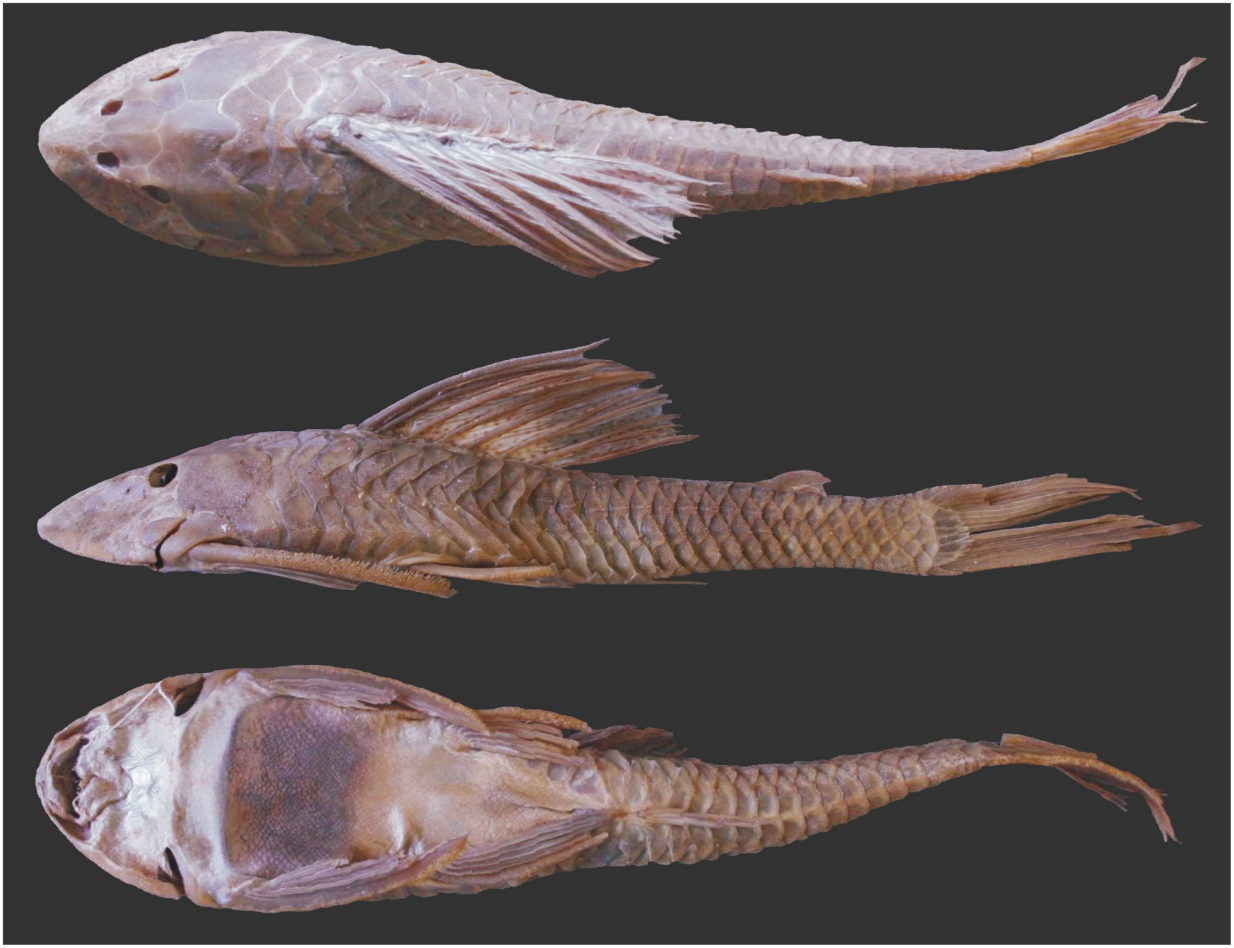
*Hypostomus subcarinatus*, MNHN A, 9575, 241.8 mm SL, holotype, Brazil, Province de Mines [state of Minas Gerais].

**Fig 2:**
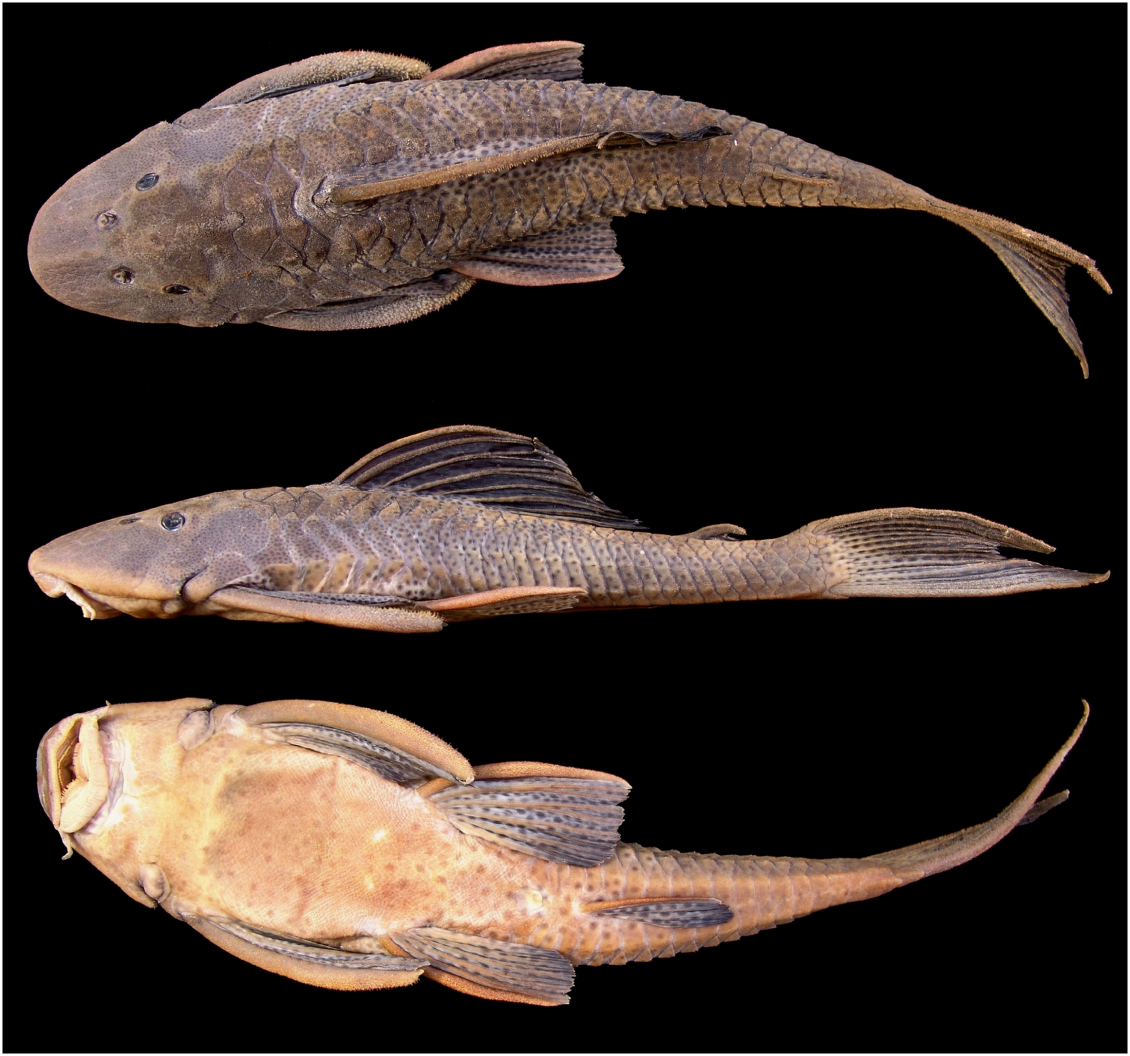
*Hypostomus subcarinatus*, MCNIP 1103, 249.5 mm SL. Lagoa da Pampulha, Belo Horizonte, Minas Gerais State, Brazil.

**Fig 3:**
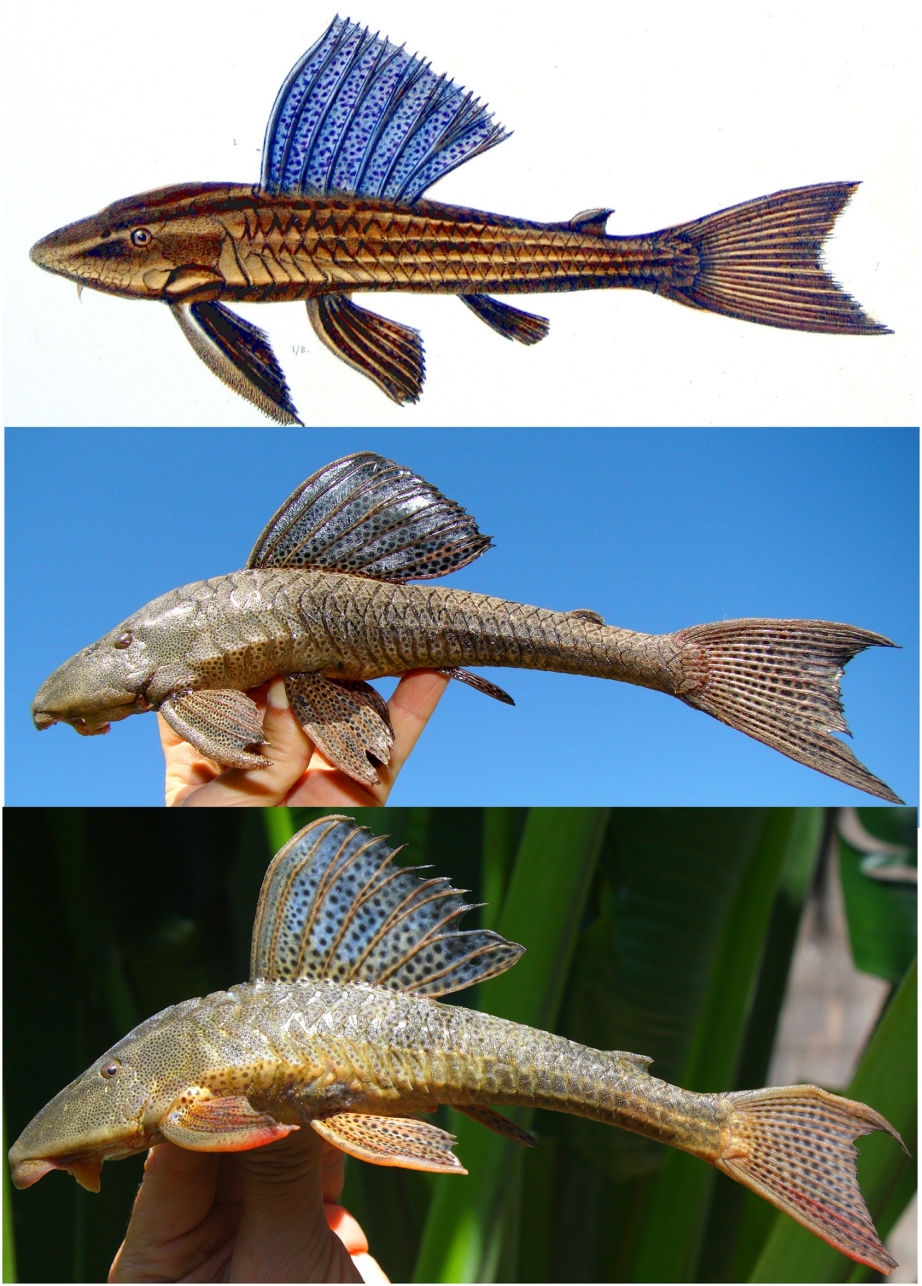
The Castelnau’s (1855) drawing of *Hypostomus subcarinatus*, MNHN A 9575, 241.8 mm SL, holotype, Brazil, Province de Mines [state of Minas Gerais], is depicted in the upper picture and compared to two live specimens photographed immediately after capture: MCNIP 1761, middle picture 227.2 mm SL and lower picture 196.5 mm SL, both from the Lagoa da Pampulha, Belo Horizonte, Minas Gerais State, Brazil.

Morphometric data in Table 1. Overall view of body in Figs 1, 2 and 3. Head moderately depressed and slightly compressed. Snout and anterior profile of head slightly pointed in dorsal view. Eye of small size, dorsolaterally positioned. Dorsal margin of orbit not raised. Greatest body width at cleithrum, narrowing from dorsal-fin region to caudal-fin origin. Dorsal profile of head convex from snout tip to vertical through interorbital region, forming angle of about 40° with ventral region of head; slightly convex from that point to dorsal-fin origin; straight from that point to caudal-peduncle end; rising to procurrent rays of dorsal fin. Ventral profile almost straight from snout tip to insertion of pelvic-fin unbranched ray; tapering slightly, straight from pelvic-fin insertion to first ventral caudal-fin procurrent ray. Anterior portion of caudal peduncle rounded with its dorsal surface compressed; posterior portion ellipsoid. Mesethmoid forming weak longitudinal bulge from snout tip to nares. Supraoccipital bone with slightly-developed median ridge and short posterior process bordered by single plate. Weak bulge originating lateral to nares, passing through supraorbital, and extending as ridge along dorsal portion of compound-pterotic. Opercle large, its horizontal length equal to distance between nares, with thin skin layer surrounding its ventral edges to subocular cheek plates. Oral disk round, moderate in size; its margins smooth. Lower lip falling short of transverse line through gill openings; ventral surface with two or three transverse dermal flaps posteriorly margining each dentary ramus; short naked area followed by larger area with numerous small papillae decreasing in size distally. Maxillary barbel moderately long, slightly larger than eye to nare distance; mostly free from lower lip. Odontodes present on anterior surface of upper lip, just below snout. Dentaries moderate to strongly angled, averaging from 90° to 100° between left and right dentary rami. Teeth viliform, bicuspid with lateral cusp smaller than mesial cusp; crown bent inward. Internally to mouth, transversal areas of short papillae bordering each premaxillary and dentary teeth rami. Median buccal papilla present and well developed.

**Table 1:** Morphometric data of the holotype plus range, mean and standard deviation (SD) of 24 non-type specimens of *Hypostomus subcarinatus*.

	holotype	range	mean	SD
Standard length (mm)	241.8	158.1–308.9	215.5	-
**Percent of SL**				
Predorsal length	35.7	33.8–37.0	35.4	1.0
Head length	29.1	26.9–29.7	28.2	0.8
Cleithral width	25.6	23.4–26.6	24.8	0.8
Head depth	15.8	15.4–17.8	17.1	0.5
Interdorsal distance	23.7	20.3–23.6	22.3	0.9
Caudal peduncle length	34.9	31.8–36.0	34.4	1.1
Caudal peduncle depth	8.3	7.1–8.4	7.7	0.3
Dorsal-spine length	30.4	22.7–33.1	27.8	2.7
Thoracic length	23.0	20.7–24.4	22.8	1.0
**Percent of head length**				
Cleithral width	87.9	84.6–92.7	87.7	2.4
Head depth	54.4	57.5–63.2	60.5	1.6
Snout length	60.3	57.4–62.4	59.8	1.4
Orbital diameter	11.8	11.1–13.7	12.5	0.7
Interobital width	38.5	33.1–39.4	37.1	1.6
Mandibular width	15.7	12.4–14.7	13.8	0.7
**Other percentages**				
Orbital diameter in snout length	19.6	17.8–23.9	20.9	1.6
Orbital diameter in interorbital length	30.7	29.5–38.4	22.7	2.9
Mandibular length in interobital length	40.7	31.4–43.8	37.2	2.6
Dorsal-spine length in predorsal length	85.1	66.3–93.5	79.1	7.3
First pectoral-fin ray length in predorsal length	75.8	70.4–79.5	73.8	2.8
Ventral caudal-fin ray length in predorsal length	88.4	91.6–108.3	97.4	4.6
Adipose-fin length in caudal peduncle depth	85.5	72.0–106.6	87.9	10.8
Caudal peduncle depth in caudal peduncle length	23.8	20.4–25.0	22.5	1.4
Mandibulary width in cleithral width	17.8	13.3–17.1	15.7	0.9
Interdorsal length in dorsal-fin base	98.3	82.6–99.1	91.9	4.2
Lower lip length in lower lip width	33.1	22.2–34.5	27.0	3.3
**Counts**			mode	
Median plates series	28	28–32	30	-
Plates bordering supraoccipital	1	1–1	1	-
Predorsal plates	3	3–3	3	-
Dorsal plates below dorsal fin bases	8	8–8	8	-
Plates between dorsal and adipose fins	9	7–9	8	-
Plates between adipose and caudal fins	6	6–7	6	-
Plates between anal and caudal fins	14	14–16	15	-
Premaxillary teeth	34	37–53	43	-
Dentary teeth	34	36–54	44	-

Body covered with five rows of dermal plates with moderately-developed odontodes, except on base of dorsal fin and small naked area on snout tip. Predorsal region with very slight median keel. Dorsal, mid-dorsal, mid-ventral, and ventral series of plates with moderate keels. Median series with weakly developed keels; bearing uninterrupted lateral line. Ventral series bent ventrally. Ventral surface of head covered with platelets, except for region behind lower lip. Abdomen covered with minute platelets in specimens larger than 110 mm SL, with exception to very small areas around pectoral- and pelvic-fin insertions (ICT-UFMG 2885). Distal portion of first anal-fin pterygiophore exposed.

Dorsal fin II,7, its origin at vertical just posterior midpoint between pectoral- and pelvic-fin insertions; first spine present as *V*-shaped spinelet. Distal margin of dorsal fin slightly convex; tip of last dorsal-fin ray falling two or three plates short of adipose-fin spine. Adipose-fin spine compressed and slightly curved inward. Pectoral fin I,6, its distal border straight. Pectoral-fin spine slightly curved ventrally, covered with moderately developed odontodes. Odontodes curved inward, more developed along distal portions of spine, particularly in larger specimens; emerging from swollen papillae. Tip of adpressed pectoral fin reaching to basal one-fourth to one-fifth of adpressed pelvic-fin unbranched ray. Pelvic fin i,5, its distal border straight to slightly convex; its adpressed unbranched ray surpassing one to two plates beyond anal-fin origin. Anal fin i,4, its tip reaching to seventh plate after its origin; its distal margin straight. Caudal fin i,14,i, its margin falcate, with ventral lobe longer than dorsal.

#### Color in alcohol

Overall ground color of dorsal and ventral regions of body and fins grayish-brown (Figs 1 and 2). Head, trunk and fins covered by numerous small dark brown spots except on lower lip. Spots very small, numerous, close together and inconspicuous in head; increasing in diameter towards posterior region of body; spots more conspicuous on fins and dorsolateral regions of trunk. Spots on ventrolateral regions of trunk usually inconspicuous. Ventral surface of body usually with faded dark spots; conspicuousness variable among specimens. All fins with many small dark spots; spots irregularly distributed on spines and either on unbranched and branched rays. Some specimens with five faded oblique dark bars on dorsum, first bar on posterior portion of head, stronger at middle of orbit, second bar at first dorsal-fin branched rays, third bar at last dorsal-fin branched ray, fourth bar at anterior region of adipose fin and fifth bar at procurrent caudal-fin rays. Ventral surface of body slightly clearer than dorsal surface.

#### Color in life

Color pattern of living specimens similar to preserved ones, except for more brownish-green background, black and more conspicuous spots and dorsal fin with blue-tan (Fig 3).

#### Sexual dimorphism

No sexual dimorphism was observed.

#### Distribution

**Fig 4:**
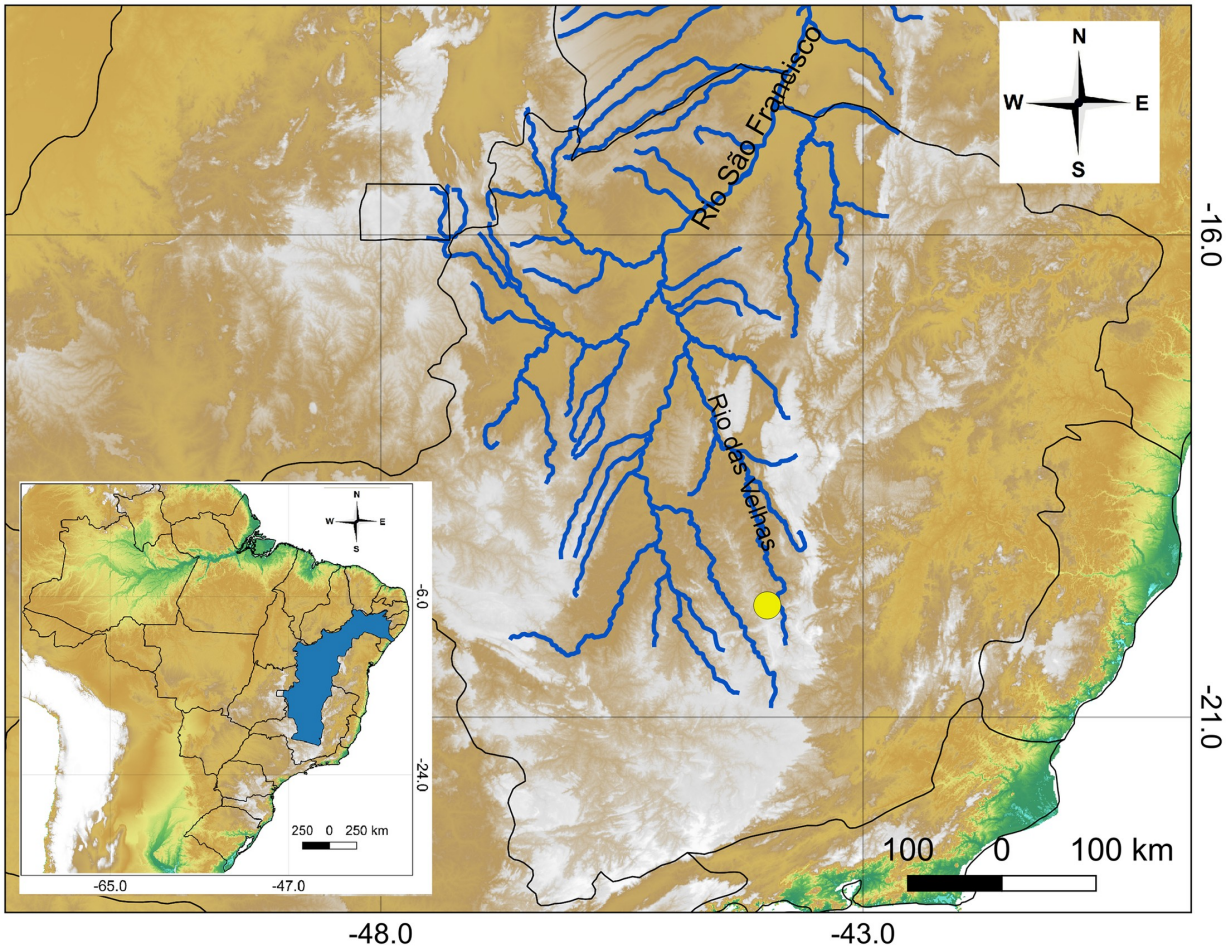
Geographical distribution of *Hypostomus subcarinatus*; (yellow circle = Lagoa da Pampulha). Blue shaded area and lines are the Rio São Francisco basin.

**Fig 5:**
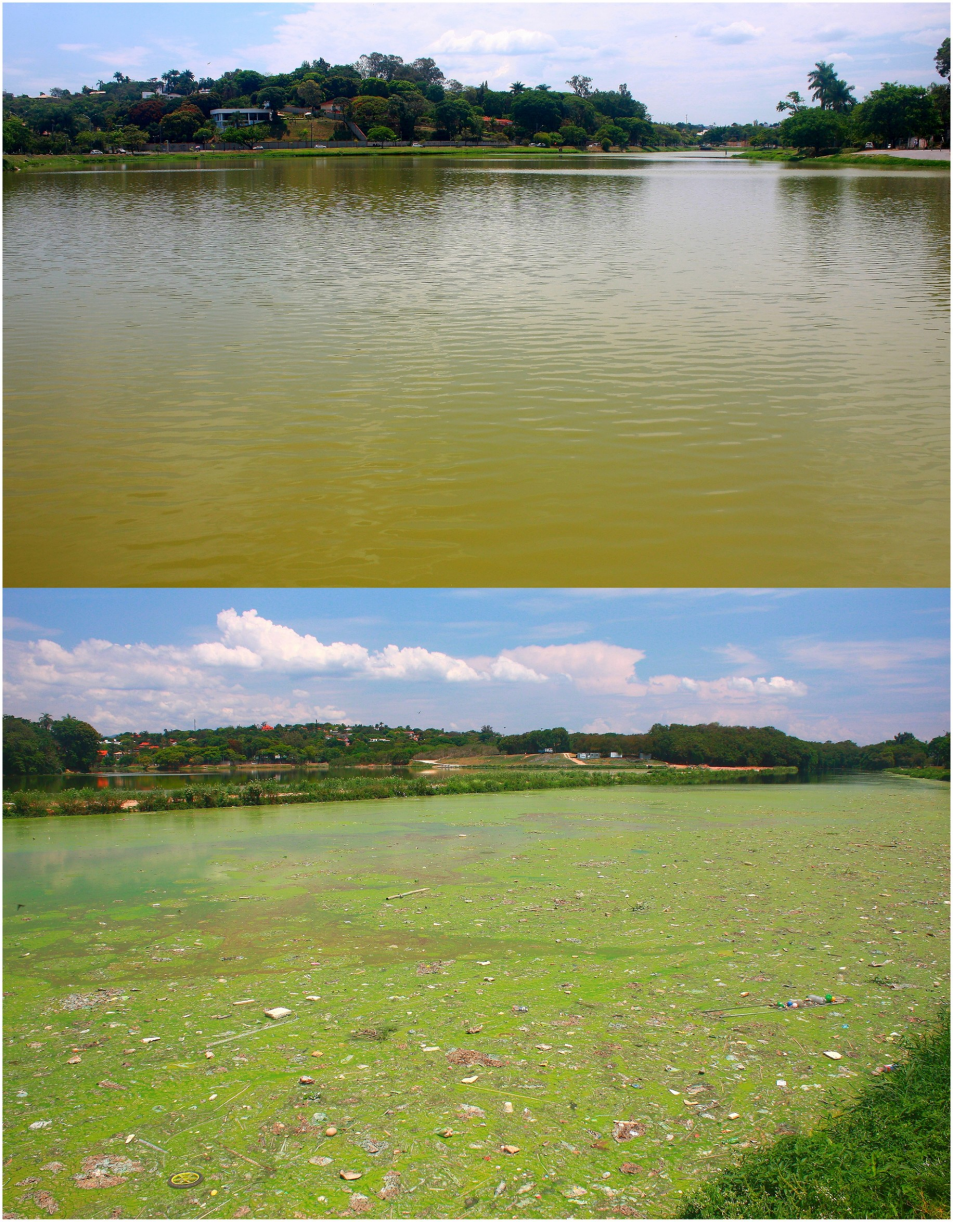
The Lagoa da Pampulha, at downtown of Belo Horizonte, Minas Gerais State, Brazil. The habitat of *Hypostomus subcarinatus*.

*Hypostomus subcarinatus* is currently known from one locality (Figs 4 and 5), the Lagoa da Pampulha, an eutrophic reservoir, in the Rio das Velhas basin, city of Belo Horizonte. The description of *Hypostomus subcarinatus* by Castelnau [1] was based on only one specimen from the ambiguous type locality “des rivières de la province des Mines”. However, Papavero [13] (p. 149–159) outlined the details of Castelnau’s itinerary, illustrating it with a map (map 12). Castelnau [14] traveled through Minas Gerais by road from the mouth to the headwaters of the Rio Paraibuna, tributary of Rio Paraíba do Sul. Then, he crossed the Rio das Mortes (upper Rio Paraná basin) at Barbacena, and its tributary the Rio Carandaí. Then, he entered to the Rio São Francisco basin, crossing the headwaters of the Rio Paraopeba, and reached the Rio das Velhas (Rio São Francisco basin) at Santa Rita (currently municipally of Nova Lima) and followed the river downstream, crossing some of its tributaries to Sabará. In the narrative of his passage through Minas Gerais, Castelnau [1,14] mentioned that he had bought fishes from local fishermen for his collection only when crossing the Rio do Peixe (left bank tributary of Rio das Velhas), but the only exact type locality cited for species from the Rio São Francisco basin is the Rio Sabará, a right bank tributary of Rio das Velhas (for *Hypostomus alatus* Castelnau, 1855: 41, and *Chalceus carpophagus* Castelnau, 1855: 68 (non Valenciennes, 1850) = *Brycon orthotaenia*). The exclusion of that locality for *H*. *subcarinatus*, just following the description of *H*. *alatus*, seems to be intentional. From Sabará, he crossed the Rio Paraopeba at São Joaquim de Bicas, the Rio Pará at Pitangui, and the Rio São Francisco at Extrema (municipality of Bom Despacho). Finally, after Patrocínio, Castelnau crossed the Rio Paranaíba (Rio Paraná basin), having passed many of its tributaries, and entered in the state of Goiás, near Catalão.

Thus, the presence of *H*. *subcarinatus* in the Lagoa da Pampulha as a result of an anthropic introduction from some unknown close or distant place seems highly improbable. The distance between this lake and the Rio das Velhas is less than 11 kilometers in a straight line. Castelnau [14], visited the village of Curral del Rei (currently, city of Belo Horizonte) and may have collected the holotype in the surroundings. It is probable that specimens of *H*. *subcarinatus* inhabit some other spots along the Rio das Velhas basin. However, until now, no specimens have been assigned to this species except for the unique holotype and those from the Lagoa da Pampulha.

#### Habitat and conservation status

*Hypostomus subcarinatus* has only been found in the Lagoa da Pampulha, a silted and polluted urban reservoir (Fig 5). The Lagoa da Pampulha was formed in 1938 to supply water to the city of Belo Horizonte. Since 1970, the reservoir has became quite eutrophic due to the dump of domestic and industrial effluents from the city, causing recurrent cyanobacteria blooms [15]. Friese *et al*. [16] found significant values of heavy metals in lake sediments. As *Hypostomus* are known to be detritivorous fishes they probably assimilate considerable proportions of those metals, as Veado *et al*. [17] found in the omnivorous *Oreochromis niloticus* in the Lagoa da Pampulha. Despite some ichthyologic survey efforts, *H*. *subcarinatus* were up to now not collected in the surroundings of the reservoir. Therefore, *H*. *subcarinatus* with an estimated Area of Occupancy (AOO) equal to the Lagoa da Pampulha area (1.96 km^2^) is herein assessed as Critically Endangered (CR) according to IUCN criteria [12], becoming the first threatened species of the genus. *Hypostomus subcarinatus* occurs sympatrically to three alien cichlids in the lake, *Coptodon rendalli*, *Oreochromis niloticus*, *Parachromis managuensis*.

## Discussion

Concerning external morphology, the most similar species to *Hypostomus subcarinatus* are the eastern Brazilian drainage dwellers *H*. *affinis*, *H*. *interruptus*, *H*. *nigrolineatus*, *H*. *punctatus* and *H*. *scabriceps*. All species have an elongate and narrow body with small to medium-sized dark spots and weak to moderate keels along lateral series of plates. Besides the blue-tan dorsal-fin, *H*. *subcarinatus* is distinguished from these congeners by being more elongate and slender than the aforementioned species and by having a longer anal-fin unbranched ray. Anal-fin unbranched ray length is almost equal or equal to snout length vs. equal to the pre-nasal length (that, is the distance from the snout tip to the anterior margin of the naris). Additionally, *H*. *subcarinatus* is distinguished from *H*. *nigrolineatus* by having unorganized dark spots versus dark spots horizontally aligned to form conspicuous stripes on laterals of trunk. Regarding the species of *Hypostomus* from the Rio São Francisco basin, the most morphologically similar species is the recently described *H*. *velhochico* Zawadzki, Oyakawa & Britski, 2017 [18]. *Hypostomus subcarinatus* is mainly differentiated from *H*. *velhochico* by attaining a larger size, having odontodes on dorsal-fin spine and on unbranched caudal-fin rays, and by having more numerous and closer to each other dark spots on head, trunk and fins.

Few papers dealt with *Hypostomus* from the Rio São Francisco basin and its taxonomy is far from being well known [18]. Most *Hypostomus* records for the basin are from dam construction monitoring programs, and not results of ecological or taxonomic citations on scientific papers. However, several specimens of *Hypostomus* are deposited in ichthyological collections, mainly in the Museu de Ciências Naturais da PUC Minas (MCNIP), ICT-UFMG, Museu Nacional, Rio de Janeiro (MNRJ) and at the Museu de Zoologia, Universidade de São Paulo (MZUSP). Except for the specimens from the Lagoa da Pampulha, *H*. *subcarinatus* were not recognized from *Hypostomus* samples at these collections.

Finding *H*. *subcarinatus* in the Lagoa da Pampulha in downtown Belo Horizonte, the third largest metropolis of Brazil with more than 5.9 millions inhabitants, was indeed a quite unexpected event. This is a fish larger than 300 mm in total length rediscovered more than 160 years after its original description and last citation. The individuals were found in the shallow, polluted urban lake, which is a significant ecological event. Some papers address ecological surveys in urban neotropical streams [19,20], but focusing fish conservation on urban neotropical artificial lake is an underestimated issue. Present findings highlight the importance that taxonomy-focused scientific surveys in such highly vulnerable water bodies, which can reveal important data to vertebrate conservation purposes. Urban lakes are frequently dragged, canalized, dried, and cleaned, to a series of reasons for human purposes. This study shows that even bad smelling urban waters as the Lagoa da Pampulha can harbor rare and endangered fish, deserving conservation management.

## Material examined

### All from Brazil, except when notices

*Hypostomus alatus*: Minas Gerais State, Rio São Francisco basin: NUP 9119, 1, 110.1 mm SL, Rio Curimataí. NUP 9829, 5, 139.0–177.4 mm SL, Rio das Velhas. NUP 9837, 4, 124.4–217.6 mm SL, Rio Cipó.

*Hypostomus ancistroides*: São Paulo State, Rio Tietê basin. LBP 2520, 2, 111.4–112.2 mm SL, Rio Tietê. MCP 28309, 1, 138.0 mm SL, Rio Piracicaba. MCP 28310, 3, 111.0–149.0 mm SL, Rio Piracicaba. MZUSP 2131, 4, 95.6–165.1 mm SL, Rio Tatuí. NUP 64, 2, 55.0–74.6 mm SL, Rio Capivara. NUP 4012, 3, 75.1–86.1 mm SL, Rio Ipanema. NUP 4016, 5, 89.1–133.6 mm SL, Rio Corumbataí.

*Hypostomus aspilogaster*: Rio Grande do Sul State, Rio Uruguai basin. ANSP 21781, 1, 204.0 mm SL, lectotype (designated by Reis *et al*., 1990), Rio Jacuí. ANSP 21782, 3, 210.6–190.0 mm SL, paralectotypes, Rio Jacuí. NUP 4355, 1, 155.0 mm SL, Rio Ibicuí da Armada.

*Hypostomus borellii*: Bolivia. Rio Paraguai basin. BMNH 1897.1.27.19, 1, 153.1 mm SL, syntype, Río Pilcomayo.

*Hypostomus boulengeri*: Mato Grosso State, Rio Paraguai basin. NUP 414, 3, 165.8–175.6 mm SL. NUP 3273, 8, 110.0–166.0 mm SL. NUP 8695, 1, 170.0 mm SL, Rio Manso. NUP 1078, 2, 210.0–220.0 mm SL, Rio Manso Reservoir. NUP 8692, 1, 190.0 mm SL, Rio Quilombo, Rio Manso basin.

*Hypostomus brevicauda*: Bahia State. BMNH 1864.1.19.16–17, 2, 189.0–196.1 mm SL, syntypes. MCP 36709, 3, 52,7–125.4 mm SL, Córrego Traíra, municipality of Camacã. MZUSP 111259, 4, 40.5–113.4 mm SL, Rio Gongogi, tributary of Rio de Contas.

*Hypostomus carinatus*: Amazonas State, Rio Amazonas basin. INPA 1198, 2, 176.7 mm SL, Rio Trombetas. INPA 2535, 1, 182.6 mm SL and INPA 2541, 1, 191.9 mm SL, Rio Uatumã.

*Hypostomus chrysostiktos*: Bahia State, ANSP 185374, 1, 166.6 mm SL, Rio Paraguaçu, Rio Paraguaçu basin.

*Hypostomus commersoni*: Uruguay. Montevideo Department. Río de La Plata basin. MNHN A.9444, 425.00 mm SL, holotype, Río de la Plata. Brazil. Santa Catarina State, Rio Uruguai basin. NUP 15804, 1, 214.0 mm SL, Rio Ijuí. NUP 16849, 168.0 mm SL, Rio Pelotas. MZUSP 107406, 1, 159.1 mm SL, Rio São Francisco, UHE Xingó-CHEESF, downstream the reservoir.

*Hypostomus delimai*: Border of the states of Tocantins and Pará, Rio Araguaia basin. NUP 11015, 1, 204.3 mm SL, unnamed stream tributary of Rio Araguaia. NUP 11016, 1, 176.7 mm SL, Rio Lontra. NUP 11017, 1, 205.5 mm SL, unnamed stream tributary of Rio Araguaia.

*Hypostomus dlouhyi*: Paraguay. Alto Paraná Department. Río Paraná basin. MHNG 2229.43, 139.5 mm SL, holotype, Río Yguazú.

*Hypostomus francisci*: Minas Gerais State. Rio São Francisco basin. MCP 14038, 1, 180.0 mm SL, Três Marias Reservoir. NUP 9940, 6, 111.0–187.1 mm SL and NUP 9945, 2, 148.6–150.7 mm SL, Rio das Velhas.

*Hypostomus garmani*: Minas Gerais State, Rio São Francisco basin. BMNH 1904.1.28.3, holotype, 209.9 mm SL. NUP 9819, 9, 87.7–204.2 mm SL; NUP 10028, 1, 78.8 mm SL and NUP 10031, 6, 136.6–170.2 mm SL, all from Rio das Velhas.

*Hypostomus jaguar*: Bahia State, Rio Paraguaçu basin. MZUSP 90870, 13, 68.8–175.6 mm SL, paratypes, Rio Paraguaçu, MZUSP 110603, 164.8 mm SL, holotype, Rio Paraguaçu. NUP 4448, 2, 126.8–152.9 mm SL, Rio Paraguaçu.

*Hypostomus johnii*: Piauí State, Rio Parnaíba basin. MCZ 7831, 1, 94.0 mm SL, syntype, Rio Poti. MCZ 7864, 2, 93.1–95.5 mm SL, syntypes, Rio Poti. NUP 12789, 1, 139.7 mm SL, Riacho Quilombo. NUP 12790, 1, 91.2 mm SL, Rio Poti.

*Hypostomus lima*: Minas Gerais State, Rio São Francisco basin. BMNH 1876.1.10, 2, 72.9–86.1 mm SL, syntypes, Lagoa Santa. NUP 5717, 4, 56.1–126.0 mm SL, Ribeirão dos Patos. NUP 5721, 2, 47.5–72.8 mm SL, Ribeirão das Minhocas. NUP 9827, 18, 81.5–181.5 mm SL, Rio São Miguel.

*Hypostomus macrops*: Minas Gerais State, Rio São Francisco basin. NUP 9831, 2, 97.7–106.8 mm SL and NUP 9832, 1, 172.6 mm SL, Rio das Velhas. NUP 9238, 1, 157.9 mm SL, Rio Curimataí.

*Hypostomus micromaculatus*: Surinam. RMNH 25483,1, 171.0 mm SL, Surinam River. RMNH 25938, 1, 166.0 mm SL.

*Hypostomus nigrolineatus*: Minas Gerais State, Rio Jequitinhonha basin. MZUSP 93743, 1, paratype, 115.7 mm SL, Rio Araçuaí, municipality of Araçuaí. MZUSP 106743, 2, 192.3–196.5 mm SL, paratypes, municipality of Padre Carvalho, Rio Vacaria. NUP 15447, 2, 162.4–212.5 mm SL, paratypes, municipality of Grão Mogol, Rio Itacambiruçu. NUP 16879, 3, 103.3–138.7 mm SL, paratypes, municipality of Itinga, Rio Araçuaí.

*Hypostomus nudiventris*: Ceará State, ANSP 69402, 56.8 mm SL, holotype and NUP 14687, 2, 78.5–100.3 mm SL, Rio Choró, municipality of Fortaleza, Northern Brazilian coastal drainages.

*Hypostomus pantherinus*: Bolivia. Beni Departament. AMNH 39946, 2, 128.2–129.5 mm SL, Rio Itenez, Rio Guaporé basin. Mato Grosso State. MCP 35962, 3, 112.8–141.2 mm SL, Rio Guaporé, Rio Madeira basin.

*Hypostomus papariae*: Rio Grande do Norte State. ANSP 69398, 94.3 mm SL, holotype, Lago Papary, Northern Brazilian coastal drainages. ANSP69399, 1, 99.1 mm SL, paratype, collected with holotype. ANSP 69400, 2, 102.7–126.6 mm SL, paratypes, Rio Choró, Northern Brazilian coastal drainages, municipality of Fortaleza. NUP 14684, 10, 54.6–104.4 mm SL, Rio Ariri, municipality of Nísia Floresta.

*Hypostomus piratatu*: Paraguay. Paraguarí Department. Río Paraguay basin. MHNG 2265.03, 214.0 mm SL, holotype, Río Paraguai.

*Hypostomus plecostomus*: Suriname. MCZ 8025, 1, 169.0 mm SL; exact locality unknown. RMNH 3102, lectotype (designated by Boeseman, 1968), 221.3 mm SL; Suriname River. ZMA 105.023, 2, 100.5–110.3 mm SL; Mama Creek, Brokopondo.

*Hypostomus punctatus*: Minas Gerais State. NUP 2605, 2, 172.0–203.0 mm SL, Rio Pomba. NUP 9670, 1, 133.3 mm SL, tributary to Rio Paraibuna, Rio Paraíba do Sul basin. NUP 14483, 1, 220.3 mm SL, Rio José Pedro, Rio Doce basin. NUP 15488, 5, 117.5–256.6 mm SL, Rio José Pedro, Rio Doce basin.

*Hypostomus pusarum*: Ceará State, Northern Brazilian coastal drainages. CAS 122225, 142.6 mm SL, holotype, Rio Ceará Mirim. CAS 122221, 4, 94.4–141.7 mm SL, paratypes. NUP 14685, 10, 64.7–180.3 mm SL, Rio Ceará Mirim, Northern Brazilian coastal drainages. Rio Grande do Norte State, Rio Piranhas-Açu basin. NUP 4795, 11, 140.0–207.0 mm SL, Rio Acauã and NUP 14683, 2, 103.1–135.0 mm SL, Rio Piranhas. Pernambuco State, Rio São Francisco basin. NUP 13973, 1, 188.0 mm SL and NUP 13974, 2, 197.5–221.7 mm SL, Itaparica reservoir, Rio São Francisco.

*Hypostomus rhantos*: Venezuela. AUM 42100, 4 of 8 paratypes, 161.5–176.8 mm SL; CAS 156859, 1, 70.5 mm SL, Río Orinoco. MCZ 68123, 1, 35.0 mm SL. Río Orinoco basin.–LBP 2185, 1, 80.2 mm SL; Río Cataniapo.

*Hypostomus subcarinatus*: Minas Gerais State, Rio São Francisco basin: MNHN A. 9575 (holotype), 241.8 mm SL, des rivière de la province des Mines [streams from the state of Minas Gerais]. Lagoa da Pampulha, tributary of Córrego da Onça, Rio das Velhas, Rio São Francisco basin: ICT-UFMG 2885, 18, 110–226.0 mm SL, 8 Jan 2002, V. Vono, B. P. Maia & L. G. M. Silva. MCNIP 1103, 7, 158.1–249 mm SL, 19°50’30”S 43°59’38”W, 30 Jan 2014, A. A. Weber & D. Gontijo. NUP 20229, 7, 164.9–248.9 mm SL, 19°50’30”S 43°59’38”W, 23 Dec 2017, I. S. Penido & T. C. Pessali. MCNIP 1761, 7, 191.1–308.8 mm SL, 19°50’30”S 43°59’38”W, 15 Apr 2016, I. S. Penido, C.H. Zawadzki, F. M. Azevedo & T. C. Pessali.

*Hypostomus tapijara*: Paraná State, Rio Ribeira de Iguape basin. NUP 863, 9, 85.9–251.3 mm SL. NUP 869, 25, 111.0–350.0 mm SL and NUP 2795, 3, 174.9–193.2 mm SL, Rio Capivari.

*Hypostomus unae*: Bahia State, Rio de Contas basin. NUP 9811, 5, 78.9–53.7 mm SL, Rio das Pedras. NUP 9814, 81.5–102.7 mm SL, Rio Oricó. MCP 41473, 10, 80.2–126.5 mm SL, Rio Preto do Costa. Rio Pardo basin. MCP 41334, 3, 55.2–120.8 mm SL, Rio Panelinha.

*Hypostomus velhochico*: Minas Gerais State. Rio São Francisco basin: MZUSP 73816, 1, 83.4 mm SL, paratype, municipality of Presidente Juscelino. NUP 12065, 92.6 mm SL, paratype, municipality of Pirapora, Rio das Velhas. NUP 12066, 1, 82.8 mm SL, paratype, municipality of Santana do Pirapama, Rio das Velhas. NUP 12067, 1, 80.2 mm SL, paratype, municipality of Santana do Pirapama, Rio das Velhas.

*Hypostomus watwata*: French Guyana. MNHN A. 8919 (lectotype of *Hypostomus verres* designated by Boeseman, 1968), 194.5 mm SL, Rio Cayenne. Guyana. BMNH 1932.11.10.31 (neotype designated by Boeseman, 1868), 261.2 mm SL, Berbice River.

*Hypostomus wuchereri*: Bahia State. BMNH1863.3.27.15, 1, syntype, 203.8 mm SL, exact locality unknown. BMNH 1852.13.12.8, 1, 127.3 mm SL, syntype, exact locality unknown.

## Supporting information

S1 Table Measures and counts. Individual measures in millimeters and counts for the holotype and 24 specimens of *Hypostomus subcarinatus*. For the proportion and percentage ranges the holotype data were not included.
(XLSX)
